# Korean childhood asthma study (KAS): a prospective, observational cohort of Korean asthmatic children

**DOI:** 10.1186/s12890-019-0829-3

**Published:** 2019-03-15

**Authors:** Dong In Suh, Dae Jin Song, Hey-Sung Baek, Meeyong Shin, Young Yoo, Ji-Won Kwon, Gwang Cheon Jang, Hyeon-Jong Yang, Eun Lee, Hwan Soo Kim, Ju-Hee Seo, Sung-Il Woo, Hyung Young Kim, Youn Ho Shin, Ju Suk Lee, Jisun Yoon, Sungsu Jung, Minkyu Han, Eunjin Eom, Jinho Yu, Woo Kyung Kim, Dae Hyun Lim, Jin Tack Kim, Woo-Sung Chang, Jeom-Kyu Lee

**Affiliations:** 1 Department of Pediatrics, Seoul National University College of Medicine, Seoul, South Korea; 2 Department of Pediatrics, Korea University College of Medicine, Seoul, South Korea; 3 Department of Pediatrics, Hallym University Kangdong Sacred Heart Hospital, Seoul, South Korea; 4 Department of Pediatrics, Soonchunhyang University School of Medicine, Bucheon, South Korea; 5 Department of Pediatrics, Korea University Anam Hospital, Seoul, South Korea; 6 Department of Pediatrics, Seoul National University Bundang Hospital, Seongnam, South Korea; 7 Department of Pediatrics, National Health Insurance Service Ilsan Hospital, Ilsan, South Korea; 8 Department of Pediatrics, Pediatric Allergy and Respiratory Center, Soonchunhyang University College of Medicine, Seoul, South Korea; 9 Department of Pediatrics, Chonnam National University Hospital, Chonnam National University Medical School, Gwangju, South Korea; 10 Department of Pediatrics, School of Medicine, The Catholic University of Korea, Bucheon St. Mary’s Hospital, Bucheon, South Korea; 11 Department of Pediatrics, Dankook University Hospital, Cheonan, South Korea; 12 Department of Pediatrics, College of Medicine, Chungbuk National University, Cheongju, South Korea; 13 Department of Pediatrics, Pusan National University Yangsan Hospital, Yangsan, South Korea; 14 Department of Pediatrics, Gangnam CHA Medical Center CHA University School of Medicine, Seoul, South Korea; 15 Department of Pediatrics, Samsung Changwon Hospital, Sungkyunkwan University School of Medicine, Changwon, South Korea; 16 Department of Pediatrics, Mediplex Sejong hospital, Incheon, South Korea; 17 Department of Pediatrics, Asan Medical Center, University of Ulsan College of Medicine, Seoul, South Korea; 18 Department of Clinical Epidemiology and Biostatistics, Asan Medical Center, Seoul, South Korea; 19 Asan Institute for Life Sciences, University of Ulsan College of Medicine, Seoul, South Korea; 20 Department of Pediatrics, Inje University Seoul Paik Hospital, Seoul, South Korea; 21 Department of Pediatrics, School of Medicine, Inha University, Incheon, South Korea; 22 Department of Pediatrics, School of Medicine, The Catholic University of Korea, Uijeongbu St. Mary’s hospital, Uijeongbu, South Korea; 23 Division of Allergy and Chronic Respiratory Diseases, Center for Biomedical Sciences, Korea National Institute of Health, Korea Centers for Disease Control and Prevention, Osong, South Korea

**Keywords:** Asthma, Child, Cluster, Cohort study, Korea, Prospective

## Abstract

**Background:**

Asthma is a syndrome composed of heterogeneous disease entities. Although it is agreed that proper asthma endo-typing and appropriate type-specific interventions are crucial in the management of asthma, little data are available regarding pediatric asthma.

**Methods:**

We designed a cluster-based, prospective, observational cohort study of asthmatic children in Korea (Korean childhood Asthma Study [KAS]). A total of 1000 Korean asthmatic children, aged from 5 to 15 years, will be enrolled at the allergy clinics of the 19 regional tertiary hospitals from August 2016 to December 2018. Physicians will verify the relevant histories of asthma and comorbid diseases, as well as airway lability from the results of spirometry and bronchial provocation tests. Questionnaires regarding subjects’ baseline characteristics and their environment, self-rating of asthma control, and laboratory tests for allergy and airway inflammation will be collected at the time of enrollment. Follow-up data regarding asthma control, lung function, and environmental questionnaires will be collected at least every 6 months to assess outcome and exacerbation-related aggravating factors. In a subgroup of subjects, peak expiratory flow rate will be monitored by communication through a mobile application during the overall study period. Cluster analysis of the initial data will be used to classify Korean pediatric asthma patients into several clusters; the exacerbation and progression of asthma will be assessed and compared among these clusters. In a subgroup of patients, big data-based deep learning analysis will be applied to predict asthma exacerbation.

**Discussion:**

Based on the assumption that asthma is heterogeneous and each subject exhibits a different subset of risk factors for asthma exacerbation, as well as a different disease progression, the KAS aims to identify several asthma clusters and their essential determinants, which are more suitable for Korean asthmatic children. Thereafter we may suggest cluster-specific strategies by focusing on subjects’ personalized aggravating factors during each exacerbation episode and by focusing on disease progression. The KAS will provide a good academic background with respect to each interventional strategy to achieve better asthma control and prognosis.

## Background

Asthma has been characterized as a chronic inflammatory airway disease with bronchial hyperresponsiveness to a variety of stimuli, as well as variable airflow obstruction that is reversible either spontaneously or with treatment [1]. Control of airway inflammation has been a mainstay of asthma treatment, particularly control of eosinophilic inflammation mediated by immunoglobulin E (IgE) production in response to a specific or non-specific stimulus [2, 3]. Asthma guidelines recommend the use of inhaled corticosteroid to control airway inflammation and to reduce episodes of asthma exacerbation that require additional systemic glucocorticoid administration and/or beta-agonist inhalation [4–6]. However, airway inflammation is not always eosinophilic, and airflow obstruction sometimes occurs without airway inflammation [7]. Therefore, asthma is now regarded as a syndrome comprising heterogeneous disease entities with variable responses to selected therapies [8].

Asthma can be classified in various ways [9]. Atopy has long been utilized as a criterion to classify pediatric asthma, and it appears to be a reasonable approach. Intrinsic asthma shows no clinical or serological evidence of IgE-mediated allergy to common environmental agents; moreover, it presents different immunopathology and lung function outcomes to extrinsic asthma [10, 11]. Atopy or high IgE level is an important determinant of asthma inception and persistence; thus, it is known as the intermediate phenotype of asthma [12]. However, simple classification into intrinsic or extrinsic asthma cannot explain various asthma phenotypes that are encountered in the clinic. In adult asthma, latent class analysis has succeeded in revealing various asthma phenotypes [13]. Furthermore, the addition of further variables regarding asthma pathophysiology can help to evaluate various asthma endotypes [14]. This approach is critically important, because asthma control and final outcome may be associated with these endotypes; they may ultimately enable the provision of a patient-tailored management plan for asthma [15].

In addition to the progress in adult studies, endotyping of pediatric asthma has now been documented in a few reports. However, most studies that have presented asthma clusters and differences in treatment responses included participants in the Severe Asthma Research Program [16–18]. Other researchers are now massively clustering clinical information [19], or working to merge cohort data (i.e., Childhood Asthma Management Program, CAMP) with genetic information [20], their results have not been replicated for the subsequent studies yet. Furthermore, ethnic and environmental factors can exhibit significant effects, but a data archiving program of Korean asthmatic children does not exist. From this perspective, a prospective study must be launched to archive clinical characteristics, environmental factors, asthma control status, allergy profile, lung function (including bronchial responsiveness, candidate biomarkers, and treatment responses), and their changes over time.

**Fig. 1:**
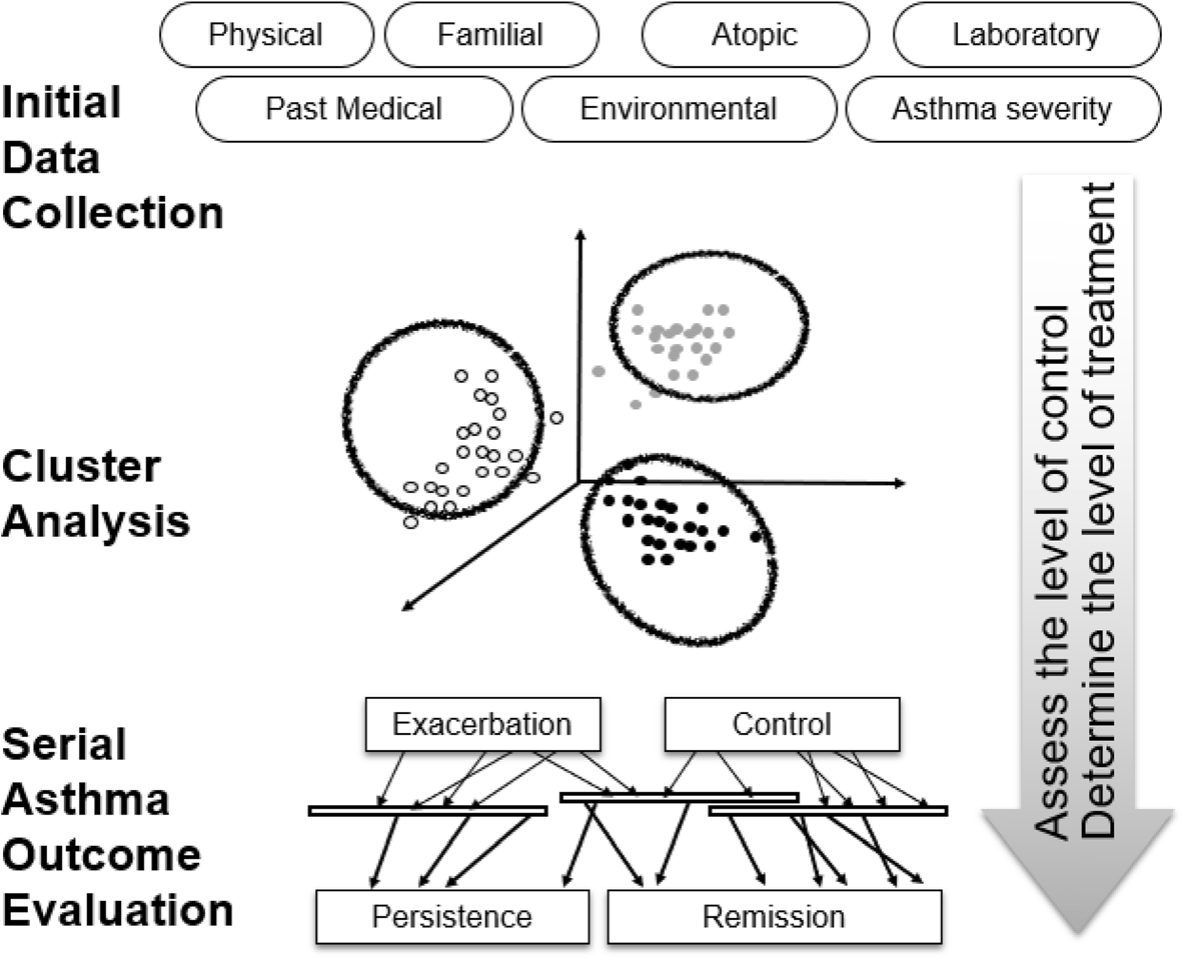
Conceptual diagram of Korean childhood Asthma Study. Hierarchical clustering of the initial data will classify pediatric asthma into several clusters; the archived data will show differences in exacerbation and asthma progression among Korean childhood asthma clusters

We designed a cluster-based, prospective, observational cohort study of asthmatic children in Korea. We will gather information regarding Korean pediatric asthma patients, classify them by using cluster analysis (include laboratory and other potential biomarkers) [21], trace their changes over time, and compare asthma exacerbation and progression between clusters (Fig. 1). We will ultimately explore factors that may help us to identify subjects’ asthma endotypes and appropriate strategy to prohibit progression to adult asthma.

## Methods/design

### Study design

**Fig. 2:**
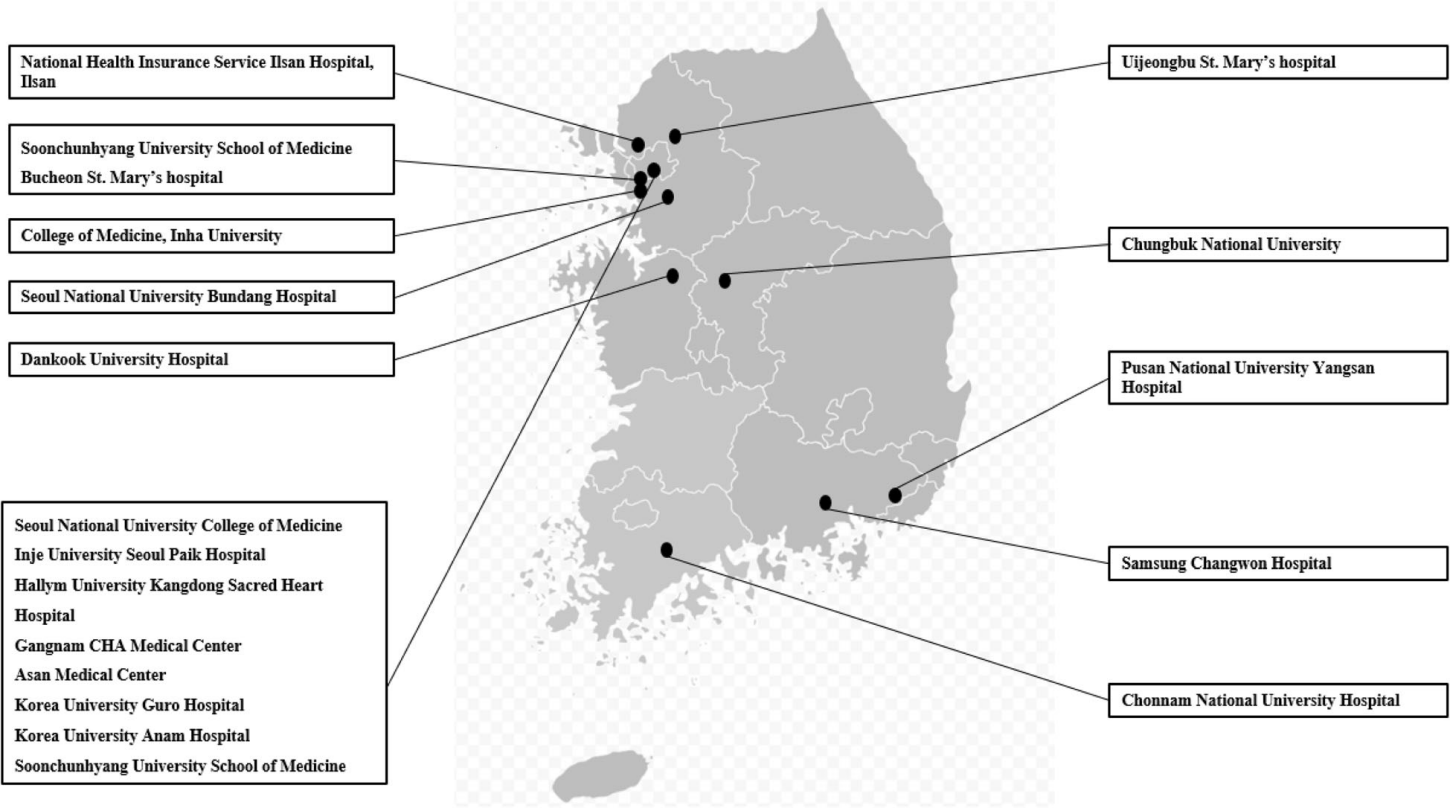
The 19 hospitals participating in the Korean childhood Asthma Study (KAS). The original shapefile of the map is from Wikimedia Commons (South_Korean_presidential_election_2007.svg licensed with Cc-by-sa-3.02012–11-23 T04:30:57Z)

KAS is an ongoing, real-world, observational prospective cohort study that involves 1000 asthmatic children at 19 centers in Korea (Fig. 2). Researchers invite subjects to participate in the study at the clinic: if they agree to participate, researchers gather baseline characteristics via a set of questionnaires, pulmonary function data including bronchial responsiveness, blood samples for allergies, and additional potential variables. In a subset of children (*n* = 400), we distributed peak-flow meters and educated caregivers regarding measurement of peak-flow, as well as reporting peak-flow results and daily symptoms through a mobile application. Subjects’ treatment responses, including levels of asthma control and episodes of exacerbation, lung function, and changes in body physics and environmental factors are evaluated at least every 6 months during regular visits. Bronchial hyperresponsiveness (BHR) and atopy are measured every 3 years during the study period. Changes in all above variables are compared between clusters at baseline and every 3 years during the study period (Table 1).

**Table 1:** Overview of data variables and collection schedule in Korean childhood Asthma Study (KAS)

	Enrollment (month)	Scheduled visits for subjects (month)			
	0	6, 12, 18, 24, 30	36	42, 48, 54, 60, 66	72
Questionnaire regarding asthma	**V**	**V**	**V**	**V**	**V**
Questionnaire regarding environment	**V**	**V**	**V**	**V**	**V**
ACT/C-ACT	**V**	**V**	**V**	**V**	**V**
Individual case record	**V**	**V**	**V**	**V**	**V**
WBC, CRP, Eosinophil count, Total IgE	**V**		**V**		**V**
Skin prick tests	**V**		**V**		**V**
FEV_1_, FVC, MMEF (pre- and post-BD)	**V**		**V**		**V**
Methacholine PC_20_ or Mannitol PD_15_	**V**		**V**		**V**
FeNO	**V**		**V**		**V**
Blood sample 15 cc for biomarker study	**V**		**V**		**V**
Mobile App for the subgroup	App installation and usage instruction	Confirmation of input and instruction			

Abbreviations: *ACT* Asthma control test, *C-ACT* Childhood asthma control test, *WBC* White blood cell, *CRP* C-reactive protein, *BD* Bronchodilator, *App* Application, *FEV*_*1*_ Forced expiratory volume in 1 s, *FVC* Forced volume vital capacity, *MMEF* Maximal mid-expiratory flow, *PC*_*20*_ Provocative concentration of methacholine causing a 20% fall in FEV_1_, *PD*_*15*_ Provocative dose of mannitol causing a 15% fall in FEV_1_, *FeNO* Fractional exhaled nitric oxide

### Study population and setting

To represent real-world asthmatic children in Korea, all children with suspected asthma are considered for study enrollment when they visit the allergy clinic at each regional center. When they meet inclusion/exclusion criteria, written consent is obtained from both patient and caregiver. Subjects are regularly followed up with various asthma treatments determined by each pediatric allergist according to the NAEPP asthma guideline [4]. When subjects are stable and show no asthma exacerbation for at least 1 month with their current asthma management, their lung function and airway inflammation are evaluated and set as their baseline values.

### Inclusion and exclusion criteria of study subjects

When subjects 5–15 years old report experiencing typical symptoms of asthma (i.e., wheezing, dyspnea, and chronic cough) within the most recent 12-month period, they are included in this cohort if they show either an elevated bronchodilator response (≥12% increase of forced expiratory volume in 1 s [FEV_1_] 15 min after inhaling 200 mcg of salbutamol) or BHR (a < 16 mg/mL dose of provocative methacholine concentration causing a 20% fall in FEV_1_ [PC_20_] or a < 635 mg dose of provocative mannitol weight causing a 15% fall in FEV_1_ [PD_15_]) [22, 23]. Subjects are excluded from this cohort if they exhibit interstitial lung diseases or pulmonary neoplasms. If subjects are already diagnosed with bronchopulmonary dysplasia or bronchiolitis obliterans, they are excluded at the enrollment stage; however, if they are newly diagnosed during follow-up, they remain in the cohort and are separately analyzed.

### Data collection

#### Questionnaires

At the time of enrollment, we ask patients and/or their caregivers to complete questionnaires regarding both the disease and the patients’ environment. The former includes asthma onset, associated allergies, past medication, level of control, compliance, familial history of asthma/allergic diseases, smoking exposure, and aggravating factors. The latter includes subjects’ housing and exposures to fine particles, yellow dusts, surrounding facilities, indoor exposure to dust mites, fungus, and cockroaches [24]. For middle school children, separate questionnaires are given to both children and their caregivers; for elementary school children, questionnaires are solely given to their caregivers. These questionnaires are given every 6 months thereafter.

#### Asthma control test

We evaluate subjects’ level of asthma control from the time of enrollment and during regular follow-up. Childhood Asthma Control Test (c-ACT) is used for subjects aged 5 to 11, and the Asthma Control Test is provided for those aged 12 and older [25, 26].

#### Physicians’ assessment

At the time of enrollment and during follow-up, subjects grade their (or their children’s) puberty status by checking an illustration of the Tanner stage grading system [27]. Physicians verify current asthma, allergic rhinitis, atopic dermatitis, and food allergies. They confirm the absence of bronchopulmonary dysplasia or bronchiolitis obliterans by reviewing subjects’ medical records and asking them detailed questions. Subjects’ asthma severity is also assessed in accordance with the Expert Panel Report 3 asthma guideline, which is used to determine the types and doses of subjects’ controller medications [4].

#### Blood tests

Blood is withdrawn to evaluate subjects’ blood cell counts, eosinophil fraction, level of total serum IgE, and amount of C-reactive protein. Additional blood samples are acquired to measure gene expression and cytokine levels, and to obtain DNA.

#### Skin prick test

At each hospital, a skin prick test on a battery of 18 inhalant allergens is performed to assess subjects’ allergen sensitization: two dust mite allergens (*D. pteronyssinus* and *D. farina*), three mold allergens (Alternaria, Aspergillus, and Cladosporium), two animal dander allergens (dog and cat), a cockroach allergen, and ten pollen allergens (oak, alder, hazel, beech, birch, rye grass, ragweed, hop, timothy, and mugwort). We use the modified method of Pepys, and the histamine and saline serve as respective positive and negative controls [28]. Subjects are defined as “sensitized” when a positive reaction, wheal size exceeding 3 mm and exceeding that of the positive control, is present for at least one allergen.

#### Lung function test and bronchial provocation test

Spirometry is performed according to international standards [29]. The forced vital capacity, FEV_1_, and the maximal mid-expiratory flow are measured before and 15 min after inhaling 2–4 puffs of salbutamol. Lung function is measured when subjects are stable with good asthma control.

Any medication that may influence lung function is withheld for 4 weeks before evaluating BHR. Methacholine challenge is conducted in accordance with ATS protocol [22]. Fresh solutions of methacholine are serially diluted in buffered saline solution at concentrations of 0, 0.0625, 0.25, 1, 4, and 16 mg/mL. Provocative concentrations of methacholine causing a 20% fall in FEV_1_ from baseline are interpolated and set as a parameter of BHR. In some cases, the mannitol provocation test may be substituted for methacholine challenge. Dry powdered mannitol (Aridol®) is sequentially inhaled, following the method suggested by Anderson et al. [23]. The provocative dose of mannitol causing a 15% fall in FEV_1_ of < 635 mg is regarded as BHR.

#### Self-assessed peak expiratory flow rate

Subjects are guided to assess their peak expiratory flow rate (PEFR) with their supplemental mini-Wright peak flow meter. They are instructed to blow the mouthpiece as quickly and as hard as possible without putting their tongue in front of the mouthpiece [29]. The best value from three big blows is recorded as their PEFR.

#### Fraction of exhaled nitric oxide

To measure subjects’ airway eosinophilic inflammation, their fraction of exhaled nitric oxide (FeNO) is measured by a hand-held device (NIOX MINO® [Aerocrine, Solna, Sweden] or NOBreath® [Bedfont Scientific, Maidstone, UK]) at every hospital. The ATS/ERS guideline is adopted for the standard procedure [30]. Briefly, FeNO is assessed during a single slow breath of at least 10 cmH_2_O against resistance and an expiratory flow rate of 50 mL/s. Exhalations are repeated up to three times and the mean is recorded as the subject’s FeNO level.

#### Mobile application

For those with Android devices, a mobile application is used to monitor subjects’ asthma exacerbation during the overall study period. Subjects are instructed to record daily symptoms (wheezing, chest tightness, dyspnea, night time symptoms, and limitation of daily activities), PEFR results, rescue medication use, unscheduled visit to a local clinic or emergency room, and need for admission until they were fully recovered.

### Data quality control

All individual case records (ICRs) are sent to the main data coordinating center in the Asan Medical Center, where a trained nurse verifies all ICR values and uploads them into the iCReaT (Internet based Clinical Research and Trial management system). The iCReaT is a web-based electronic data capture system developed and operated by a government organization, the Korea Centers for Disease Control and Prevention. Data entry to iCReaT is restricted to main center personnel. Another data manager regularly oversees the data input process and monitors the data integrity by scanning for typological errors and missing values, as well as by mining outliers and reviewing ICRs and source documents. The authors formed a data management committee composed of key researchers, data managers, and the coordinator in the main center. The committee monitors the entire process biannually, reviews queries received, discusses plans to secure the data integrity, and provides feedback to each researcher with relevant issues.

### Outcome assessments

Follow-up data regarding asthma outcome are obtained from the physicians’ record and laboratory results from each institution, as well as from questionnaires from patients/caregivers. Supplemental information imbedded to the national Health Insurance Review and Assessment service (HIRA) system is assessed to evaluate medical facility utilization among the enrolled institutions.

Asthma exacerbations are defined as episodes of progressive increase in shortness of breath, cough, wheezing, chest tightness, or a combination of these symptoms [31]; here, we practically define them as asthma attacks that result in medical interventions, including hospitalization, unscheduled visits (physician or emergency department), or prescription of oral corticosteroids (in patients who are not already on regular oral corticosteroids) [32]. Symptoms that completely disappear immediately after a single dose of inhaled salbutamol are not considered exacerbation. In the subset of subjects with mobile applications, asthma exacerbation is also defined as reduction of PEFR by more than 20% from the individual maximum level in the presence of asthma symptoms.

Uncontrolled asthma is defined as at least one of the following, in accordance with modified ERS/ATS guidelines [33]: Poor symptom control of ACT or c-ACT < 20; two or more severe exacerbations requiring systemic corticosteroid for more than 3 days in the previous year; at least one serious exacerbation requiring hospitalization, ICU stay, or mechanical ventilation in the previous year; and/or airflow limitation of a FEV_1_ < 80% after bronchodilator.

Severe asthma is defined according to ERS/ATS guidelines as asthma that requires treatment with suggested medications for GINA steps 4–5 asthma for the previous year, or systemic corticosteroid for ≥50% of the previous year to prevent it from becoming “uncontrolled,” or asthma that remains “uncontrolled” despite such therapy [33].

Clinical asthma remission is a primary outcome, and it is defined as the absence of asthma symptoms for ≥2 years and no asthma medication use for ≥2 years [34]. Asthma persistence is defined as the presence of asthma symptoms within 1 year or the use of any asthma medication in the previous year.

Laboratory changes in lung function, BHR, and atopic sensitization, according to the asthma clusters, are our primary interests. In terms of lung function, percentage predicted values are designated as comparing parameters. Changes of more than two doubling doses in PC_20_ or PD_15_ are interpreted as significant [35]. Negative or positive conversion among a panel of allergens is judged to be significant.

## Current status

**Fig. 3:**
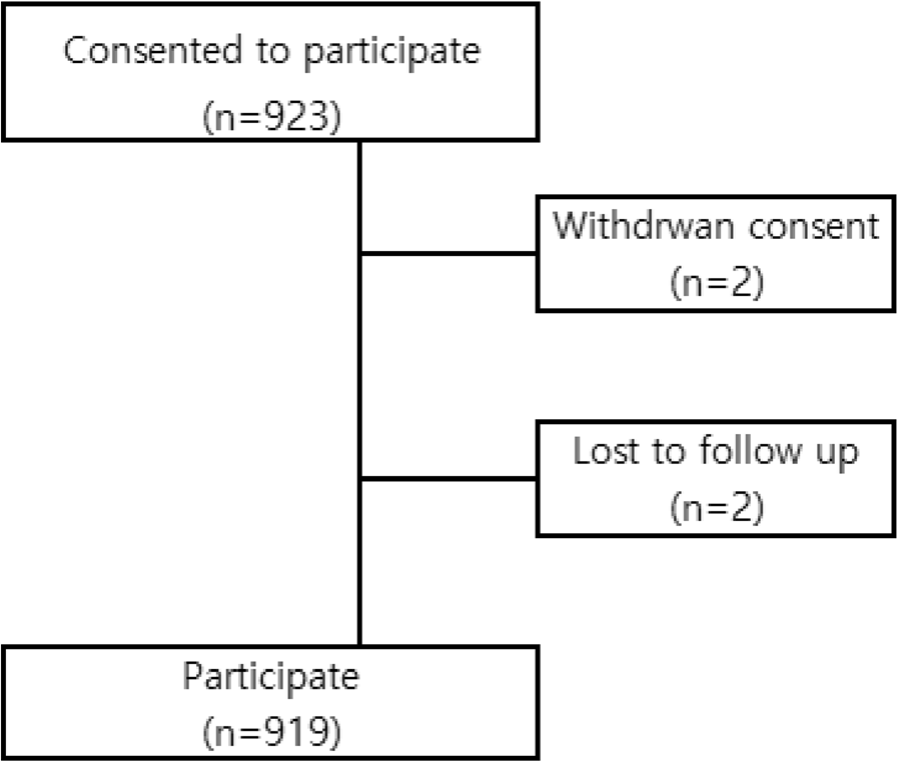
Current status of Korean childhood Asthma Study (KAS). A total of 923 asthmatic children have consented to participate in the study. Of these, two withdrew their consent and another two were lost to follow-up. Currently, the study cohort includes 919 children

As of August 2018, a total of 923 asthmatic children were enrolled in KAS. Among them, two have withdrawn from the study and two were lost to follow up. Thus, 919 children are currently enrolled in KAS (Fig. 3).

## Statistical analysis

Cluster analysis will be used via hierarchical clustering algorithm with Ward’s minimum variance method. Hierarchical cluster analysis generally comprises cluster formation by grouping closely related individuals in an individual manner. Based on the measure of the distance between the clusters, pairs are merged with each other at each step; ultimately, “n” objects are grouped together to form a cluster. Dendrograms will be used to determine the number of clusters. To test for differences between clusters, ANOVA and χ^2^ tests will be performed. All above analyses will be conducted by using SAS version 9.4 (SAS Institute Inc., Cary, NC, USA). In the subset of subjects with the mobile application, a big data-based deep learning analysis will be conducted to predict asthma exacerbation based on the spatiotemporal correlation between the asthmatic patient’s medical records, daily PEFR data, and environmental factors (including daily air pollution, pollen, and meteorological data).

## Ethical considerations

Ethical approval has been granted by the Institutional Ethics Committee of each participant institution. The study was approved by the Institutional Review Boards of Asan Medical Center (IRB No. 2016–0914), Seoul National University Hospital (IRB No. 1607–165-779), Pusan National University Yangsan Hospital (IRB No. 05–2016-121), Inha University Hospital (IRB No. 2016–07–016-008), Seoul National University Bundang Hospital (IRB No. 10–2017-036), Chonnam National University Hospital (IRB No. 2017–201), Korea University Anam Hospital (IRB No. 2015 AN 0310), Soonchunhyang University Hospital Seoul (IRB No. 2017–01–011-002), Bucheon St. Mary’s Hospital (IRB No. HC16SNMI0056), Sungkyunkwan University Samsung Changwon Hospital (IRB No. 2017–02–006-001), Kangdong Sacred Heart Hospital (IRB No. 2016–12–007-001), The Catholic University of Korea, Uijeongbu St. Mary’s Hospital (IRB No. UC16ONMI0113), Chungbuk National University Hospital (IRB No. 2016–09-003), Dankook University Hospital (IRB No. 2017–02-013), Korea University Guro Hospital (IRB No. 2016GR0336), Seoul Paik Hopsital, Inje University (IRB No. 2016–314), Gangnam CHA Medical Center (IRB No. GCI-16-37), National Health Insurance Service Ilsan Hospital (IRB No. NHIMC 2017–02-008), Soonchunhyang University School of Medicine, Bucheon (IRB No 2016–08–007-009).

## Discussion

We have launched a prospective observational disease cohort for asthmatic children in Korea. Based on the assumption that asthma is heterogeneous, the KAS gathers demographic, clinical, environmental, and laboratory variables that might affect asthma severity, and endeavors to determine its exacerbation and progression. Children’s asthma subtypes and their subset of risk factors of asthma exacerbation can subsequently be inferred by cluster analysis. Because this study archives both cross-sectional and longitudinal data, we can also evaluate relationships among variables and their changes over time. We can focus on asthma in a very specific group of children, including either adolescent or severe asthmatic patients. The KAS aims to eventually suggest a personalized strategy to achieve better asthma control and best prognosis.

Prior to enrollment, we exclude subjects with a prior history of BPD or BO. However, we do not exclude subjects who are eventually diagnosed with BPD or BO from the ongoing KAS study, because they are not easily discernable from childhood asthma at baseline and they may constitute a subset of childhood asthma patients in the real world [36].

The primary goal of the KAS is to archive information regarding the clinical and environmental characteristics of Korean asthmatic children, along with their pulmonary function and allergy status. Changes in these variables over time are also of interest. The authors hope to evaluate the following topics by analyzing the archived data:

1. Asthma clusters in Korean children
2. Nationwide distribution of asthma clusters and regional characteristics of Korean childhood asthma
3. Patterns of airway microbiome across asthma clusters
4. Comparisons of microRNA expression patterns across asthma clusters as exploratory biomarkers
5. Characteristics of severe asthma in Korean children
6. Characteristics of asthma in adolescents, compared with asthma in younger children
7. Characteristics of specific asthma phenotypes (e.g., mold-sensitized asthmatic children) in a subgroup of the study population
8. Changes in asthma severity over time and their determinants
9. Changes in lung function and BHR over time, with particular regard to asthma clusters
10. Differences in asthma outcome and treatment response among clusters
11. Environmental risk factors of asthma exacerbation with respect to asthma clusters
12. Impact of patient-tailored environmental intervention on asthma control as a spin-off trial for a small number of selected subjects
13. Determinants and prognostic factors of asthma remission in Korean children

Based on the assumption that asthma is heterogeneous and that each subject will exhibit a different subset of risk factors for asthma exacerbation and progression, the KAS first aims to identify several asthma clusters and their essential determinants, which are more applicable to Korean asthmatic children. Thereafter, our analysis may suggest cluster-specific strategies by focusing on subjects’ personalized aggravating factors during each exacerbation episode. The KAS will provide a good academic background to understand asthma pathophysiology in children and will help to identify individualized risk factors with respect to asthma exacerbation and progression.
